# Supplementary material for: Metabolomics Provide Sensitive Insights into the Impacts of Low Level Environmental Contamination on Fish Health—A Pilot Study
Source: Metabolites. 2020 Jan 6;10(1):24. doi: 10.3390/metabo10010024 (PMC7022837; doi:10.3390/metabo10010024)
Supplement: Supplementary file 1 [file metabolites-10-00024-s001.zip › metabolites-667614-supplementary.pdf]

Metabolomics Provide Sensitive Insights into the Impacts of Low Level Environmental Contamination on Fish Health – a Pilot Study

Sara M. Long *

Dedreia L. Tull

David P. De Souza

Konstantinos A. Kouremenos

Saravanan Dayalan

Malcolm J. McConville

Kathryn L. Hassell

Vincent J. Pettigrove

Marthe Monique Gagnon

* Corresponding author: Sara Long, Aquatic Environmental Stress Research Group, School of Science, RMIT-University, PO Box 71, Bundoora, 3083, Victoria, Australia.

Email address: sara.long@rmit.edu.au

Methods

Site Description

In the southeast of the Bay, the rural town of Sorrento is relatively clean and free of contamination, as no industrial discharges occur in the area. Similarly, St. Leonards has no industrial activities and low-density development nearby. In addition, these sites experience the most flushing as they are located close to the entrance of Port Phillip Bay to the ocean (Bass Strait). Sorrento and St Leonards were identified as 'rural sites' for this study. On the eastern side of the Bay, Mordialloc hosts dense residential developments as well as being the discharge site of a channel draining upstream agricultural and industrial areas. In the northernmost area, Hobsons Bay is located adjacent to the City of Melbourne which has the busiest container shipping port in Australia and is also where the Yarra River (the major river that flows through the City of Melbourne, with a catchment of > 4,000 km^2) drains into Hobsons Bay. Finally, Corio Bay is in the westernmost side of the Bay, next to the City of Greater Geelong and a large oil refinery. Hence Mordialloc, Hobson Bay and Corio Bay were identified as 'urbanised sites' for this study

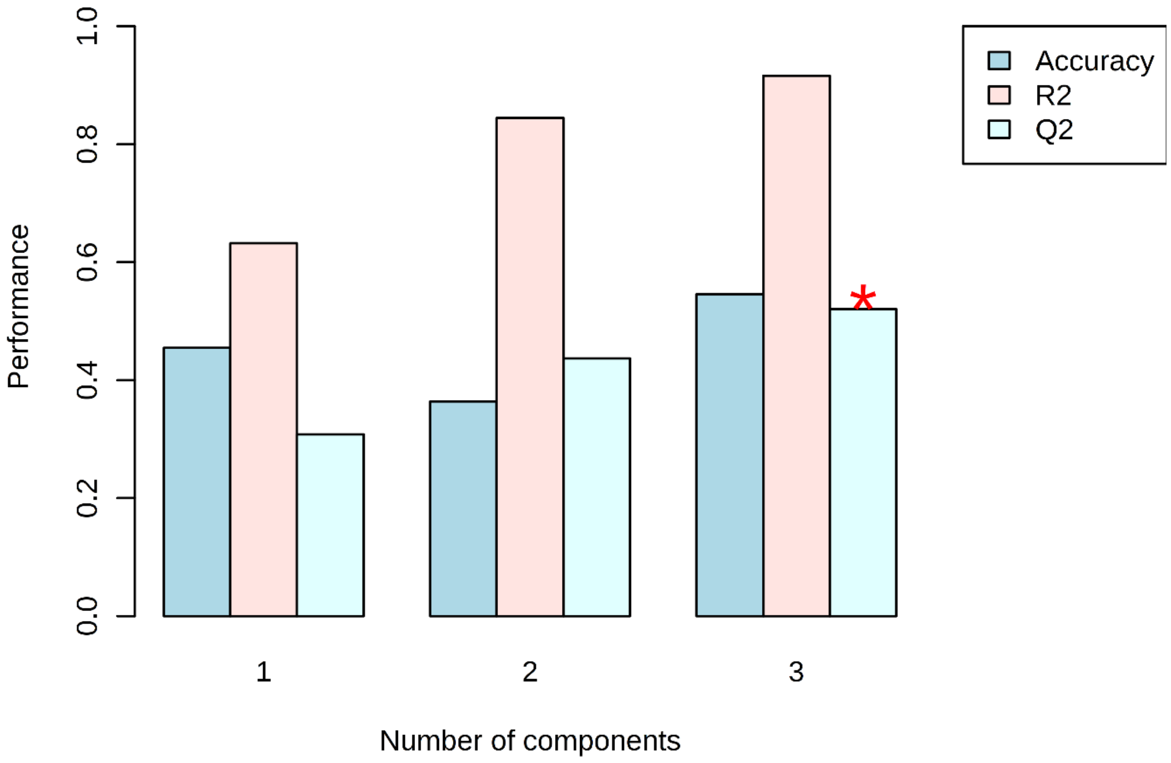

Figure S1. Cross-validation graph of PLS-DA for polar metabolites, according to Szymańska et al. [63].

Table S1. Cross-validation details of PLS-DA for polar metabolites, according to Szymańska et al. [63].

PLS-DA cross validation details:

Measure	1 comps	2 comps	3 comps
Accuracy	0.45455	0.36364	0.54545
R2	0.63205	0.84467	0.91604
Q2	0.30782	0.43687	0.52062

Table S2. Significant and trending polar metabolites (from ANOVA) detected in livers of female sand flathead *(Platycephalus bassensis)* collected from sites around Port Phillip Bay, Victoria, Australia in February-March 2015. Metabolites were considered significant if the FDR adjusted p value was <0.05. Metabolites were considered trending towards significance if FDR adjusted p value was <0.1 but >0.05 and p value was < 0.05.

**Metabolite**	***p* value**	**FDR adjusted *p* value**	**Comparison**
Lactic Acid	1.24E-05	0.000729	Significant
Oxalic acid	6.43E-05	0.001898	Significant
Proline 4-hydroxyl	0.000116	0.001949	Significant
L-Alanine	0.000132	0.001949	Significant
L-Threonine	0.000176	0.001982	Significant
3-Phosphoglyceric acid	0.000202	0.001982	Significant
L-Tyrosine	0.000936	0.007233	Significant
Urea	0.000981	0.007233	Significant
Norleucine	0.003645	0.023347	Significant
Glycine	0.003957	0.023347	Significant
L-Aspartic acid	0.005841	0.029729	Significant
Taurine	0.006047	0.029729	Significant
Phosphoenolpyruvate	0.007879	0.035374	Significant
L-Lysine	0.008394	0.035374	Significant
L-Histidine	0.009231	0.036308	Significant
D-Glucose	0.012685	0.046775	Significant
Succinic acid	0.019906	0.067805	Trending
Citric acid	0.020686	0.067805	Trending
L-Ornithine	0.022536	0.069241	Trending
D-Gluconic Acid	0.023471	0.069241	Trending
Maleic acid	0.027081	0.073311	Trending
L-Phenylalanine	0.027336	0.073311	Trending
Glycerol-2-phosphate	0.029695	0.074144	Trending
L-Tryptophan	0.03016	0.074144	Trending
L-Methionine	0.03464	0.081532	Trending
Sorbitol	0.035929	0.081532	Trending
Hypoxanthine	0.039622	0.086582	Trending

Table S3. Polar metabolites in livers of female sand flathead (*Platycephalus bassensis*) following Fishers LSD post hoc comparison. Values in bold show significantly different metabolites between the comparisons (FDR adjusted $p < 0.05$), NS = not significantly different.

Metabolite	Comparison						
	St. Leonards v Sorrento	Corio Bay v Sorrento	Mordialloc v Sorrento	Hobsons Bay v Sorrento	Corio Bay v St. Leonards	Mordialloc v St. Leonards	Hobsons Bay v St. Leonards
3-phosphoglyceric acid	NS	**< 0.05**	**< 0.05**	**< 0.05**	**< 0.05**	**< 0.05**	**< 0.05**
Glucose	**< 0.05**	NS	**< 0.05**	NS	**< 0.05**	NS	**< 0.05**
Glycine	**< 0.05**	**< 0.05**	**< 0.05**	NS	NS	NS	**< 0.05**
Lactic acid	**< 0.05**	**< 0.05**	**< 0.05**	**< 0.05**	**< 0.05**	**< 0.05**	NS
L-Alanine	**< 0.05**	**< 0.05**	**< 0.05**	**< 0.05**	**< 0.05**	**< 0.05**	NS
L-Aspartic acid	NS	NS	**< 0.05**	NS	NS	**< 0.05**	NS
L-Histidine	**< 0.05**	**< 0.05**	**< 0.05**	**< 0.05**	NS	NS	NS
L-Lysine	NS	**< 0.05**	**< 0.05**	**< 0.05**	NS	NS	NS
L-Threonine	**< 0.05**	**< 0.05**	**< 0.05**	**< 0.05**	NS	NS	NS
L-Tyrosine	**< 0.05**	**< 0.05**	**< 0.05**	**< 0.05**	**< 0.05**	NS	NS
Norleucine	**< 0.05**	**< 0.05**	**< 0.05**	NS	NS	NS	**< 0.05**
Oxalic acid	**< 0.05**	**< 0.05**	**< 0.05**	**< 0.05**	NS	NS	NS
Phosphoenol pyruvate	NS	**< 0.05**	**< 0.05**	**< 0.05**	**< 0.05**	**< 0.05**	NS
Proline 4 hydroxyl	**< 0.05**	**< 0.05**	**< 0.05**	**< 0.05**	NS	NS	NS
Taurine	**< 0.05**	NS	**< 0.05**	NS	**< 0.05**	NS	**< 0.05**
Urea	**< 0.05**	**< 0.05**	**< 0.05**	NS	NS	NS	**< 0.05**

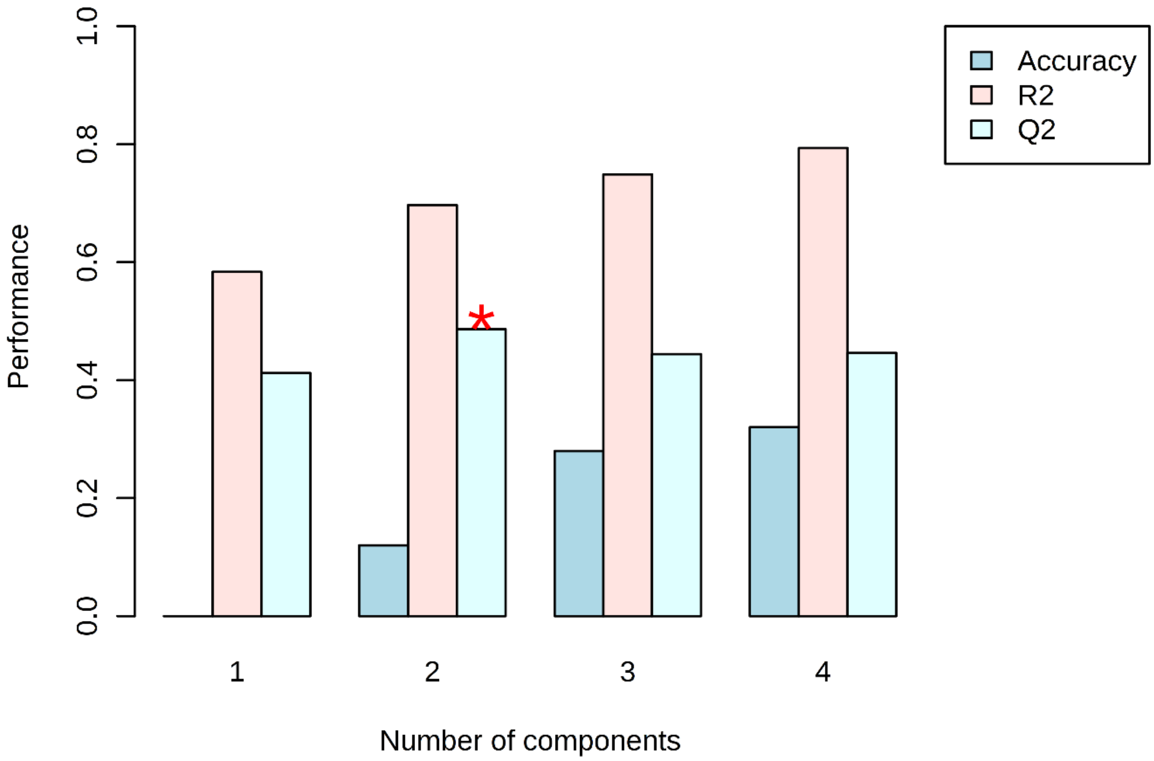

Figure S3. Cross-validation graph of PLS-DA for free fatty acid metabolites, according to Szymańska et al. [63].

Table S4. Cross-validation details of PLS-DA for free fatty acid metabolites, according to Szymańska et al. [63].

PLS-DA cross validation details:

Measure	1 comps	2 comps	3 comps	4 comps
Accuracy	0.0	0.12	0.28	0.32
R2	0.58349	0.69686	0.74871	0.79381
Q2	0.41195	0.48636	0.44416	0.44579

Table S5. Significant and trending free fatty acid metabolites from ANOVA detected in livers of female sand flathead (*Platycephalus bassensis*) collected from different sites around Port Phillip Bay, Victoria, Australia in February-March 2015. Metabolites were considered significant if the FDR adjusted p value was <0.05. Metabolites were considered trending towards significance if FDR adjusted p value was <0.1 but >0.05 and p value was < 0.05.

Metabolite	p value	FDR adjusted p value	Comparison
C18:3	0.00207	0.032373	Significant
C21:0	0.002398	0.032373	Significant
C15:0	0.008616	0.061134	Trending
C22:0	0.009057	0.061134	Trending
C17:0	0.015188	0.082013	Trending

Table S6. Free fatty acid metabolites in livers of female sand flathead *(Platycephalus bassensis)* following Fishers LSD post hoc comparison. Values in bold show statistically significantly different metabolites ($p < 0.05$).

**Metabolite**	**Comparison**						
	Corio Bay v Sorrento	Mordialloc v Sorrento	Hobsons Bay v Sorrento	Corio Bay v St. Leonards	Hobsons Bay v St. Leonards	Mordialloc v Corio Bay	St. Leonards v Mordialloc
C21:0[a]	**< 0.05**	**< 0.05**	**< 0.05**	**< 0.05**	**< 0.05**	**< 0.05**	NS
C18:3[b]	**< 0.05**	**< 0.05**	**< 0.05**	**< 0.05**	**< 0.05**	NS	**< 0.05**

[a] – saturated fatty acid

[b] – unsaturated fatty acid

Table S7. Mean temperature (°C) and total phytoplankton counts (cells/L) at sites in Port Phillip Bay, Summer 2014/2015 (Paula Sardina, EPA Victoria)

Site	Number of sampling events	Temperature °C (Range)	Total Phytoplankton cells/L (SD)
Sorrento	1	18.8	884,005
St Leonards	1	20.6	NM
Corio Bay	4	19.2 (17.1 – 21.7)	230,457 (153384)
Mordialloc	2	18.7 (17.1 – 20.4)	1,090,057 (528884)
Hobsons Bay	4	19.5 (17.6 – 22.4)	1,623,374 (1525082)

NM – not measured

Figure S2. Boxplots showing abundance of significant (FDR adjusted p value < 0.05) polar (a) or free fatty acid (b) metabolites in livers of sand flathead (*Platycephalus bassensis*) sampled at sites around Port Phillip Bay, Victoria, Australia.

Figure S2a

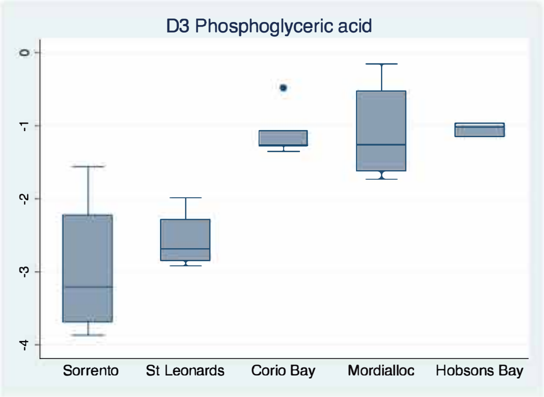
D3 Phosphoglyceric acid
0
-1
-2
-3
-4
Sorrento
St Leonards
Corio Bay
Mordialloc
Hobsons Bay

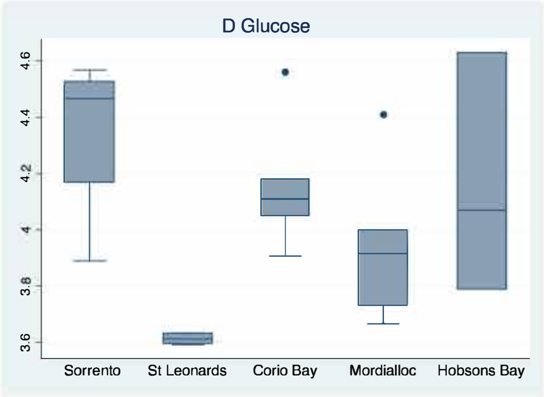
D Glucose
4.6
4.4
4.2
4
3.8
3.6
Sorrento
St Leonards
Corio Bay
Mordialloc
Hobsons Bay

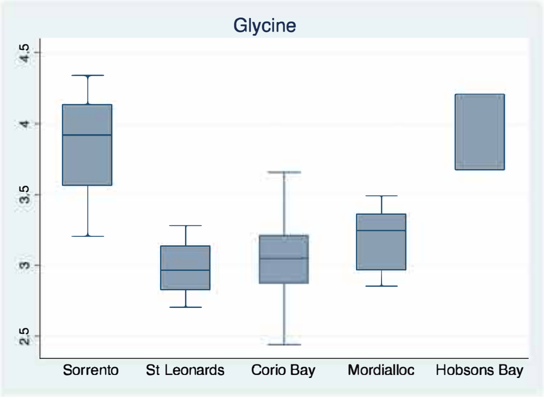
Glycine
4.5
4
3.5
3
2.5
Sorrento
St Leonards
Corio Bay
Mordialloc
Hobsons Bay

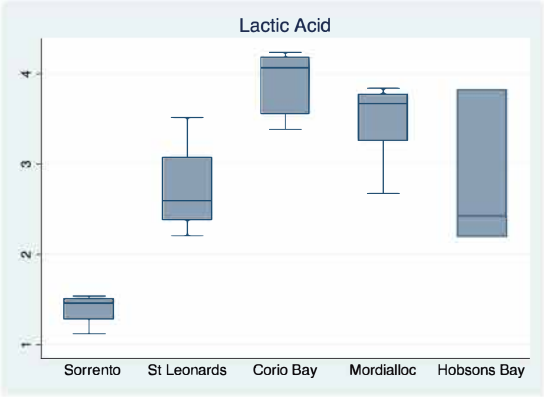
Lactic Acid
4
3
2
1
Sorrento
St Leonards
Corio Bay
Mordialloc
Hobsons Bay

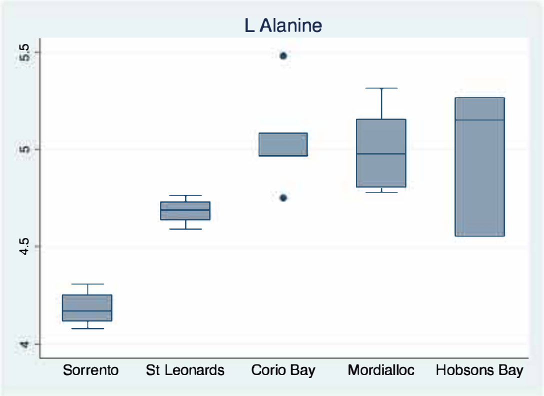
L Alanine
5.5
5
4.5
4
Sorrento
St Leonards
Corio Bay
Mordialloc
Hobsons Bay

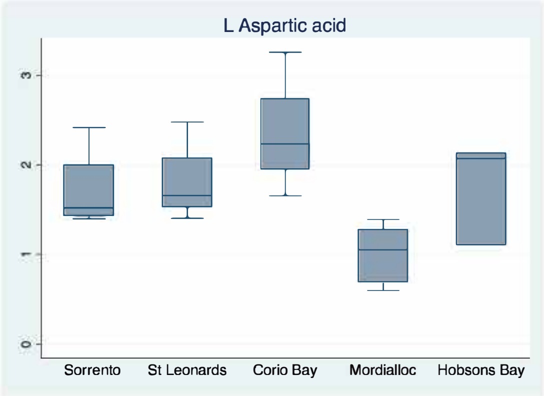
L Aspartic acid
3
2
1
0
Sorrento
St Leonards
Corio Bay
Mordialloc
Hobsons Bay

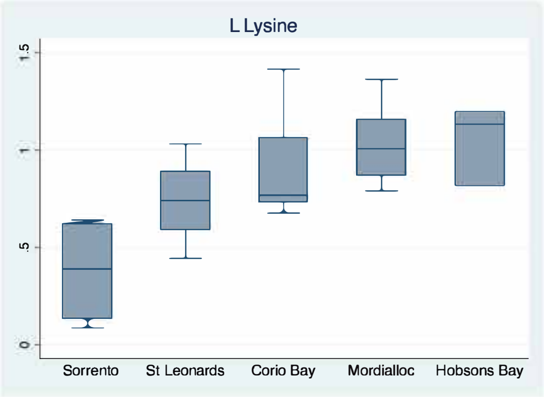
L Lysine
0
.5
1
1.5
Sorrento
St Leonards
Corio Bay
Mordialloc
Hobsons Bay

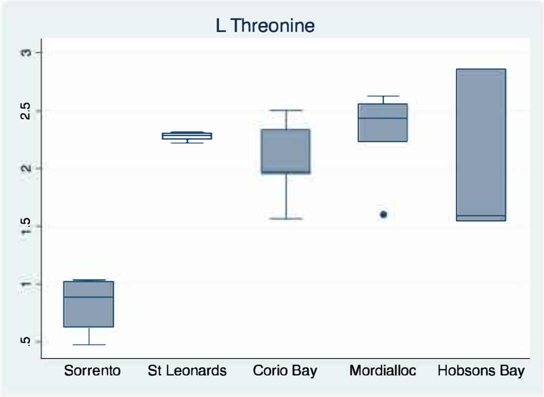
L Threonine
.5
1
1.5
2
2.5
3
Sorrento
St Leonards
Corio Bay
Mordialloc
Hobsons Bay

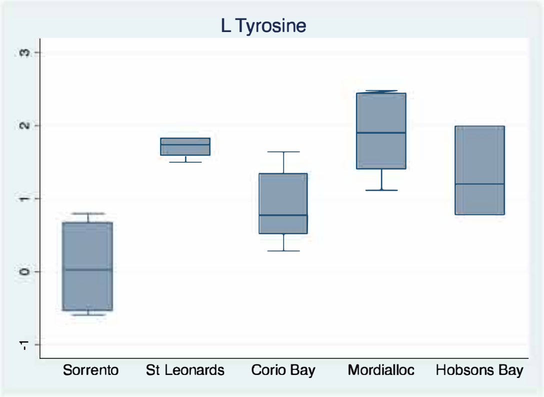
L Tyrosine
-1
0
1
2
3
Sorrento
St Leonards
Corio Bay
Mordialloc
Hobsons Bay

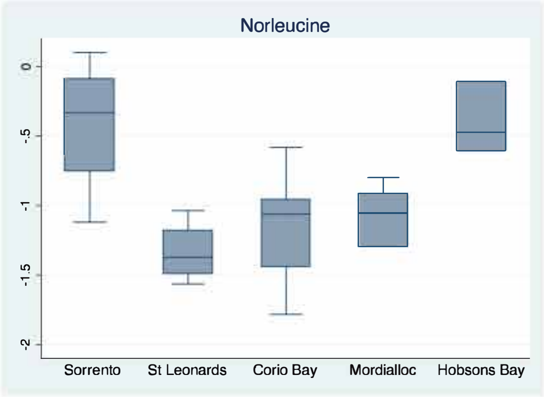
Norleucine
-2
-1.5
-1
-.5
0
Sorrento
St Leonards
Corio Bay
Mordialloc
Hobsons Bay

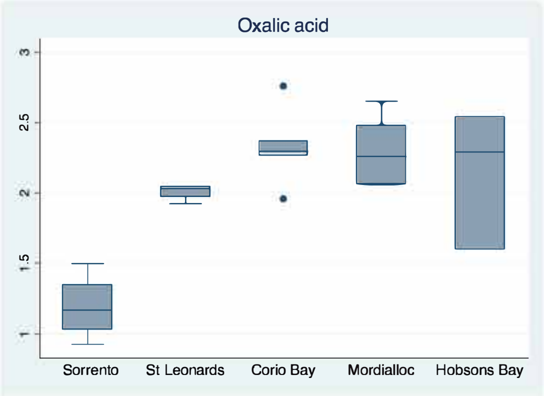
Oxalic acid
1
1.5
2
2.5
3
Sorrento
St Leonards
Corio Bay
Mordialloc
Hobsons Bay

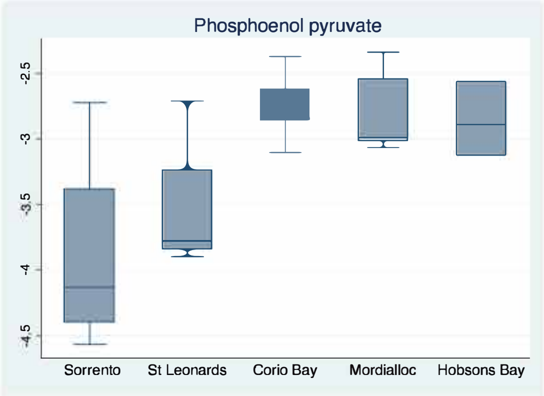
Phosphoenol pyruvate
-4.5
-4
-3.5
-3
-2.5
Sorrento
St Leonards
Corio Bay
Mordialloc
Hobsons Bay

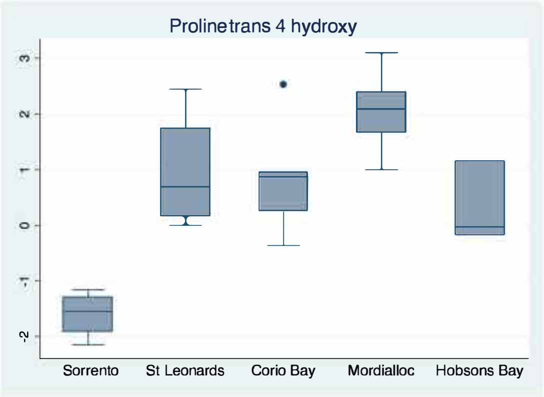
Prolinetrans 4 hydroxy
3
2
1
0
-1
-2
Sorrento
St Leonards
Corio Bay
Mordialloc
Hobsons Bay

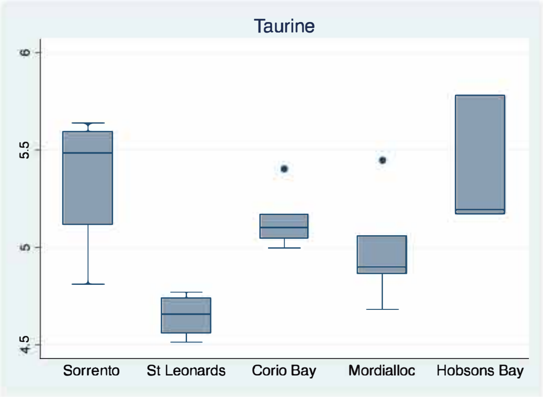
Taurine
6
5.5
5
4.5
Sorrento
St Leonards
Corio Bay
Mordialloc
Hobsons Bay

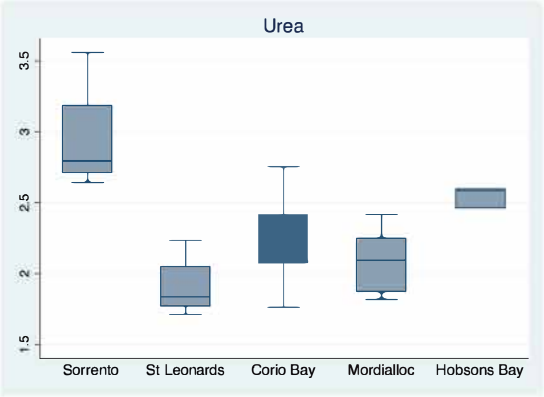
Urea
3.5
3
2.5
2
1.5
Sorrento
St Leonards
Corio Bay
Mordialloc
Hobsons Bay

Figure 2b

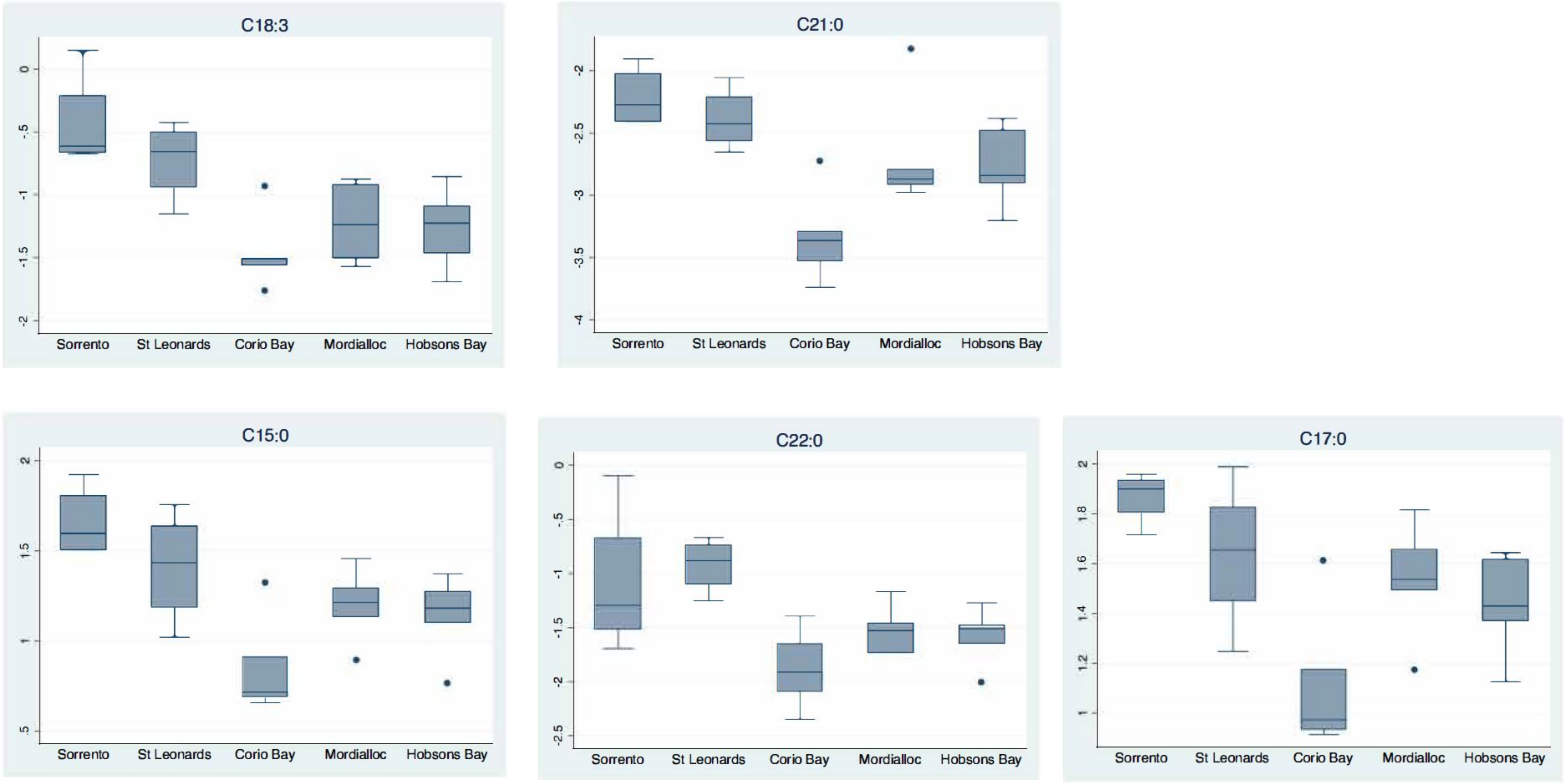
C18:3
0
-.5
-1
-1.5
-2
Sorrento
St Leonards
Corio Bay
Mordialloc
Hobsons Bay
C21:0
-2
-2.5
-3
-3.5
-4
Sorrento
St Leonards
Corio Bay
Mordialloc
Hobsons Bay
C15:0
2
1.5
1
.5
Sorrento
St Leonards
Corio Bay
Mordialloc
Hobsons Bay
C22:0
0
-.5
-1
-1.5
-2
-2.5
Sorrento
St Leonards
Corio Bay
Mordialloc
Hobsons Bay
C17:0
2
1.8
1.6
1.4
1.2
1
Sorrento
St Leonards
Corio Bay
Mordialloc
Hobsons Bay
